# Monthly Summary

**Published:** 1900-01

**Authors:** 


### Monthly Summary.

HOW TO REMOVE A PIN CEMENTED TO A ROOT.—I asked this question of a professional friend recently, and he replied, "Extract the root and split it open." I thought this a little too heroic practice, and suggested a milder one. I had a practical case in hand, a patient who had broken a cemented pin at the junction of a common pivot crown with an upper right lateral root.

It occurred to me that a strong alkali ought to dissolve the phosphoric acid of the cement, and thus cause the disintegration of the filling. So I went home and threw the pivot crown, which was a part of the metallic pin, into a bottle of strong aqua ammonia and let it remain over night. Upon removing it, found the cement a complete mush, and easily removed along with the pin also. My patient called soon after, when, with a small abscess tubular knife in the engine hand-piece, I cut the cement around the pin as far up as was expedient, and then with a fine pointed hatchet excavator proceeded to finish the operation, applying the aqua ammonia to decompose the remaining cement, and protecting the gum underneath with a piece of rubber dam covered with a napkin, stopping occasionally to rinse the mouth with warm water. After ten or fifteen minutes' manipulation, my efforts were rewarded by a slight movement of the pin, when I seized the end of it with a small pair of pliers and with rotary motion removed it.

I believe that oxyphosphate fillings last longer in mouths where there is an acid reaction of the saliva, and are less permanent where an alkaline condition prevails. This afforded me the hint, and the result was not unexpected.—William B. Mead, in *Cosmos*.

---

COMPRESSED CORK AND ITS USES.—Cork, as everyone knows, is one of the best non-conductors of heat or sound. That it has not been more widely used in building is due chiefly to the difficulty of obtaining it in an unadulterated form.

A product called cork tiling has recently been placed upon the market, which is made of what is known to the trade as "virgin cork," ground, compressed, and otherwise treated by a patented process, and which is free from the cement and glue usually employed to hold the particles together. We are informed that tiles made of this pure, compressed cork form an admirable flooring, which, besides being noiseless, water-proof, warm, and germ-proof, is capable of withstanding hard usage. By varying the degree of compression and modifying the manufacturing process slightly, sheets of cork different in color and density are obtained, which, when sawed and finished in the form of panels, can be used for wainscoting alone, or in connection with cork-tile floors. Cork compressed into sheets and sawed to the size and thickness desired constitutes a very efficient pully covering. It is said that a pulley covered or lagged with compressed cork will transmit from 50 to 60 per cent. more power with the same tension of belt than one having oniy a smooth iron surface.—Scientific American.

---

POINTS IN FAVOR OF THE USE OF ALCOHOL AND THEIR REFUTATION.—Dr. Beinfait examines point by point the various objections to total abstinence, and reaches the following conclusions:

1. Is alcohol a digestive? No, its digestion produces a passive excitation; interrupts the proper action of the stomach, because alcohol acts as an anesthetic after having irritated the walls of this action of the gastric juice.

2. Is alcohol an appetizer? No; it produces an excitation of the stomach which causes a sensation taken for hunger.

3. Is alcohol a food? No; it does not correspond to the definition of a food, and the heat that it seems to produce does not serve as actual warmth.

4. Is alcohol heating? No; it causes a flow of the blood to the skin and the lowering of temperature.

5. Is alcohol a stimulant? In no case, either physical or mental.

6. Is alcohol a protector against contagion? No; it predisposes the body to contagion.

7. Can we live without alcohol? This idea that we cannot live without alcohol is a prejudice that numerous facts contradict.

8. Is alcohol good for children? It should never be given children.

9. Does alcohol increase longevity? According to reliable statistics, alcohol diminishes longevity.—St. Louis Medical and Surgical Journal.

---

AN EVIL AND ITS REMEDY.—As we note the ever-increasing number of so-called "dental parlors," and see the columns of the daily papers disfigured by the advertisements of the disreputable practitioners of dentistry, it becomes more and more evident that the evil of dental quackery is rapidly increasing, and we are impressed with the idea that a remedy should be found to check its undue growth. It cannot be wholly eliminated, for while time lasts and men inhabit the earth, just so long will the unthinking and the credulous fall a prey to those who are unscrupulous and dishonest. So long as is inherent in the human heart the desire to get something for nothing, the man who offers to meet that want will secure patronage. But we believe that the growth of this evil as it relates to the profession may be checked very materially by insisting that our dental colleges demand of their matriculates a higher standard of preliminary education than is now generally required, and that throughout the entire college course of study they endeavor to impress on their students the value of the knowledge they are imparting and the absolute necessity for maintaining only clean and honorable methods of practice. Let only graduates of reputable colleges come before State boards of examiners, and make it necessary for all to do so; take the appointment of State board membership out of politics; make the college course extend over four years of nine months each; in short, raise the entire plane of the scheme of dental education, and we believe the percentage of those who resort to disreputable methods of practice will be reduced to a minimum. Legislation will not make men good, but education may.—Editorial in Pacific Stomatological Gazette.

---

PARALYSIS FOLLOWING GENERAL ANÆSTHESIA.—William M. Leszynsky ("Medical Record," October 21, 1899) says: The paralysis produced by pressure during anæsthesia is preventable in every instance. As a rule it affects the upper extremities. The serious attention of the surgeon should be directed to this class of cases. It is occasioned by the following conditions:

1. Prolonged elevation and extension of the arms, allowing the head of the humerus to make pressure on the brachial plexus.
2. Metal clamps or tight straps over the shoulders or lower extremities.
3. Extension of the head toward one side, thus stretching the nerve trunks and rendering them still more susceptible to pressure.
4. Placing of the arm under the head and retaining it in the same position.
5. Permitting the arm or leg to hang over the edge of the table, thus making pressure upon some individual nerve.
6. The elbows of the anesthetizer resting heavily in the clavicular region, pressing upon the brachial plexus.—*Chicago Clinic*

---

NEW METHOD OF TREATMENT OF WOUNDS.—Schleich, in his book on the treatment of wounds which has just appeared, has attempted to simplify the methods of miner surgery so as to make the general practitioner independent of what he calls the monopolization and centralization of surgery. He disputes the assertion that bacteria are the chief source of infection. He looks upon them as the accompaniments of uncleanliness of which their presence is sufficient proof, but he rejects the idea that they are the cause of sepsis and pyemia for the reason that these diseases occur in the presence ot very different forms of bacteria. He supports his ideas by the citation of other illustrations. For example, characteristic fevers follow the ingestion of spoiled meat and fish although under these circumstances only small numbers of the ordinary streptococci and staphylococci can be shown to be present. Schleich insists that the operator should disinfect himself immediately after contact with infectious material, so that at any time he may be looked upon as nearly aseptic. Hand brushes he describes as universal labyrinths for grease and dirt. He rejects all sorts of chemical methods of purification partly because they do not destroy the bacteria and partly because they can not be thoroughly carried out. He advises a mechanical cleansing calculated to wash away the bacteria. He uses a "marble soap" which is made of a special alkali (steral) and contains marble-dust to take the place of brushes. After the hands are washed they are smeared with wax paste to close the ducts of the sweat- and fat-glands which no method is capable of disinfecting. In the treatment of wounds he has small regard for antiseptics which can only control to a limited extent the development of bacteria. He advocates an early and wide incision in suppurative processes and tam ponade of the wound with gauze. He rejects all drains and uses silk instead of catgut, because he does not believe that the latter can be perfectly sterilized. He boils his silk and immerses it in gelatin to preserve it from infection. The gelatin is melted off with hot water at the time of use. He lays stress upon the necessity of introducing "homogenous substances between the edges of infected wounds. If this can be accomplished such wounds will heal in the same manner as healthy cut surfaces. Glutol, a dry and sterile formalin-gelatin, fulfills this requirement.—*Medicat Progress.*

